# Cytokine Profiling of Exudates from Periapical Lesions and the Efficacy of CXCL10 as a Healing Marker

**DOI:** 10.3390/pathogens14101013

**Published:** 2025-10-07

**Authors:** Kazuhisa Ouhara, Yuri Taniguchi, Ruoqi Zhai, Katsuhiro Takeda, Ryousuke Fujimori, Naoya Kuwahara, Shoya Ueda, Yitong Hou, Nomi Honoka, Masaru Shimizu, Shoko Kono, Tomoyuki Iwata, Shinji Matsuda, Noriyoshi Mizuno

**Affiliations:** 1 Department of Periodontal Medicine, Graduate School of Biomedical and Health Sciences, Hiroshima University, 1-2-3 Kasumi, Minami-ku, Hiroshima 734-8553, Japan; 2 Department of Biological Endodontics, Graduate School of Biomedical and Health Sciences, Hiroshima University, 1-2-3 Kasumi, Minami-ku, Hiroshima 734-8553, Japan; 3 Program of Oral Health Sciences, Department of Public Oral Health, Graduate School of Biomedical and Health Sciences, Hiroshima University, 1-2-3 Kasumi, Minami-ku, Hiroshima 734-8553, Japan

**Keywords:** C-X-C motif chemokine ligand 10, CXCL10 (IP10), periapical lesion, apical periodontitis, immune response

## Abstract

This study aimed to evaluate cytokine profiling in a periapical lesion to provide a rationale for future treatment strategies for periapical lesions. Thirteen samples of exudative fluid were collected from such a lesion directly through the root canal. Cytokine profiling was performed using the Bio-Plex system. CXCL10 (C-X-C motif chemokine ligand 10, IP10) was found to be elevated in apical exudates of patients exhibiting favorable healing. To evaluate the role of CXCL10 in cell migration, a Transwell assay was conducted using bone marrow-derived mononuclear cells (BMMCs). Different types of cytokines were detected from the samples of periapical lesion at the initial visit. However, cytokine production varied across patient samples. Release of interleukin (IL)-1β, IL-2, IL-4, IL-5, IL-6, IL-8, IL-10, IL-12, IL-13, IL-17, granulocyte colony-stimulating factor (G-CSF), granulocyte-macrophage colony-stimulating factor (GM-CSF), interferon gamma (IFN-γ), monocyte chemoattractant protein (MCP)-1, macrophage inflammatory protein (MIP)-1α, MIP-1β, and tumor necrosis factor (TNF)-α showed differential expression. Comparison of cytokine profiles indicated that cytokine production was variable before and after root canal treatment. In vitro, CXCL10 significantly improved BMMC migration in a dose-dependent manner, supporting clinical findings that elevated CXCL10 levels are associated with favorable healing in apical lesions. Although this study was limited by the small sample size and exploratory design, the cytokine profile of periapical lesions may be useful for assessing the condition of periapical lesions and modulating the immune response to bacterial infection.

## 1. Introduction

Apical periodontitis (AP) is an inflammatory disease characterized by periapical tissue injury to the affected teeth caused by microbial infection, often requiring root canal treatment (RCT) to control infection and eliminate inflammation. A meta-analysis investigating the global prevalence of AP showed that half of the adult population worldwide has AP, whereas the frequency of AP in root-filled teeth is as high as 39% [1]. Failed healing of the lesion after RCT prompts patients to seek further treatment. Furthermore, it has been reported that transmission of bacteria to distant organs may contribute to the exacerbation of various systemic diseases. AP has been associated with a range of systemic conditions, including atherosclerosis, type 2 diabetes, chronic kidney disease, and rheumatoid arthritis, largely due to its capacity to induce low-grade systemic inflammation [2]. Refractory AP (RAP) is chronic AP that persists after repeated routine RCT. Although multiple biological factors, such as intraradicular and extraradicular microbial infections and exogenous root canal filling materials, can lead to persistence of periapical lesions after RCT [3], the most common etiology of RAP is uncontrolled pathogen infection, posing a challenge to clinical therapy [4,5]. Accumulating evidence has shown that complex interactions between pathogenic microorganisms and the host immune response are important in the pathogenesis of RAP.

RCT combined with antimicrobial agents to remove the source of infection is considered the first-line treatment of these conditions in addition to basic RCT; however, when lesions become large, surgical treatment, such as root-end resection, is selected over conservative treatment. Nonetheless, in recent years, surgical treatment has become difficult with patients undergoing treatment for systemic diseases. In addition, conservative treatment for a prolonged period of time may not result in healing. Therefore, a more comprehensive understanding of the specific mechanisms of pathogen-modulated host cell responses is required to determine potential therapeutic targets to control infection and inflammation, promote tissue repair, and provide new insights into the prevention and treatment of RAP.

Osteoblasts have traditionally been thought of as cells involved in bone formation, but in recent years, they have also been implicated in immune regulation by producing cytokines in response to inflammatory stimuli [6]. CXCL10 (C-X-C motif chemokine ligand 10, IP10), in particular, has attracted attention as a chemokine that induces migration of Th1 cells and immune cells in inflammatory tissues and can also be produced by osteoblasts themselves [7].

CXCL10 is a member of the subfamily of inducible IFN-γ and non-ELR CXC chemokines, and it signals through CXCR3, a common receptor for IFN-γ–inducible chemokines (CXCL9, CXCL10, and CXCL11). The fundamental role of CXCL10/CXCR3 interaction has been well characterized in chronic Th1-inflammatory diseases. In inflamed tissues, CXCL10 recruits Th1 cells and upregulates the production of IFN-γ. In turn, IFN-γ stimulates CXCL10 expression in various cell types, resulting in a positive feedback to amplify CXCL10 and Th1 responses [8]. CXCL10 recruits CD4+ T cells and promotes them to produce RANKL, and then RANKL induces CXCL10 expression and osteoclast differentiation from its precursors [9].

This study aimed to elucidate the local expression of CXCL10 and its functional significance in AP, particularly its ability to mobilize immune cells, by combining patient-derived clinical data with in vitro cell migration experiments. In particular, we evaluated the significance of CXCL10 as a pathological biomarker and therapeutic target. We focused on the potential local production of CXCL10 that may involve osteoblasts and its involvement in the maintenance or repair of lesions via chemokine activity. Further, we focused on CXCL10 in patient-derived leachate from root tip lesions and analyzed the correlation between its concentration and the state of the lesion. Functional analysis was performed using a cell migration assay to clarify the effect of CXCL10 on immune cell mobilization.

## 2. Materials and Methods

### 2.1. Sample Collection

Overall, 13 patients (7 women and 6 men; mean age: 61.38 ± 11.91 years) aged ≥20 years, who had visited the Hiroshima University Hospital between 2012 and 2013, were included in this study (Table 1). Patients were diagnosed with one of the following conditions: acute AP, radicular cyst, or chronic AP. Participants who had received antibiotics in the preceding 3 months; patients with uncontrolled systemic diseases, such as diabetes mellitus, HIV infection, liver disease, hypertension, hyperlipidemia; and patients who had received radiation therapy, chemotherapy for cancer treatment, or immunosuppressant drugs were excluded from the study. The experimental protocol was approved by the Ethics Committee of Hiroshima University (approval No. E2011-0398). All participants provided their informed consent prior to the start of the study.

Exudates derived from AP samples were collected following a standardized protocol. First, rubber dam isolation was performed according to the standard procedure for RCT to clearly isolate the affected tooth. After removal of the temporary restoration, 50 µL of exudate was aspirated from the apical region using a root canal syringe. The collected sample was then diluted in a 50 µL of buffer solution containing protease inhibitors and stored at −80 °C until further analysis. Exudate collection was conducted at three time points: at the initiation of treatment, during the course of treatment, and the time when sterilization was confirmed by bacterial testing.

### 2.2. Comprehensive Analysis of Cytokine Production in Apical Exudate

Cytokine concentrations in exudates derived from AP were measured using the Bio-Plex^®^ system (Human Cytokine G1 27 Plex kit, #M500KCAF0Y, Bio-Rad Laboratories, Inc., Hercules, CA, USA), following the manufacturer’s instructions and a previous report [10]. The collected exudate samples were incubated for one hour with bead solutions conjugated with capture antibodies. After the beads were washed, detection antibodies were added, followed by a second 1-h incubation. Following a final washing step, the expression of inflammatory cytokines was analyzed using the Bio-Plex^®^ system (Bio-Rad). The panel of target cytokines included interleukin (IL)-1β, IL-1ra, IL-2, IL-4, IL-5, IL-6, IL-7, IL-8, IL-9, IL-10, IL-12(p70), IL-13, IL-15, IL-17, Eotaxin, bFGF, granulocyte colony-stimulating factor (G-CSF), granulocyte-macrophage colony-stimulating factor (GM-CSF), interferon gamma (IFN-γ), CXCL10, monocyte chemoattractant protein (MCP)-1, macrophage inflammatory protein (MIP)-1α, MIP-1β, Regulated on Activation, Normal T cell Expressed and Secreted (RANTES), tumor necrosis factor (TNF)-α, and vascular endothelial growth factor (VEGF).

### 2.3. Collection of Bone Marrow Mononuclear Cells

Bone marrow-derived mononuclear cells (BMMCs) were collected from the femurs and tibias of C57BL/6J Jcl mice by density gradient centrifugation with Histopaque-1083 (#10831, Sigma-Aldrich, St. Louis, MO, USA) in complete alpha-modified Eagle’s minimum essential medium (#M4526, Sigma-Aldrich), containing 10% fetal bovine serum (#A5256701, Invitrogen, Thermo Fisher Scientific, Waltham, MA, USA), L-glutamine, (#25030081, Sigma-Aldrich), and antibiotics (penicillin, streptomycin, and gentamicin, #P4333, Invitrogen, Thermo Fisher Scientific).

### 2.4. Migration Assay

The BMMC migration assay was performed using Transwell migration assay kits with 24-well plates containing an 8.0 µm pore membrane insert (#3422, Corning, NY, USA). BMMCs (1.0 × 10^5^ cells/well) were seeded in the upper layer. The lower layer contained CXCL10 (0, 1, 10, 100 ng/mL, #266-IP-050, R&D systems, Minneapolis, MN, USA). Following incubation in serum-free α-MEM at 37 °C under 5% CO_2_ for 12 h, non-migrated cells were eliminated using a cotton swab, and migrated cells were fixed with 4% paraformaldehyde (#163-20145, Cell Signaling Technology, Danvers, MA, USA) for 15 min for cell counting.

### 2.5. Statistical Analysis

All experiments were performed independently at least three times. Data are expressed as the mean ± standard deviation (SD). The Shapiro–Wilk test was conducted for each data set to evaluate the normality of the distribution. Comparisons between the two groups were made using two-tailed unpaired Student’s *t*-tests. *p*-values < 0.05 were considered statistically significant.

## 3. Results

### 3.1. Cytokine Profiling of Periapical Exudate of Patients

**Figure 1:**
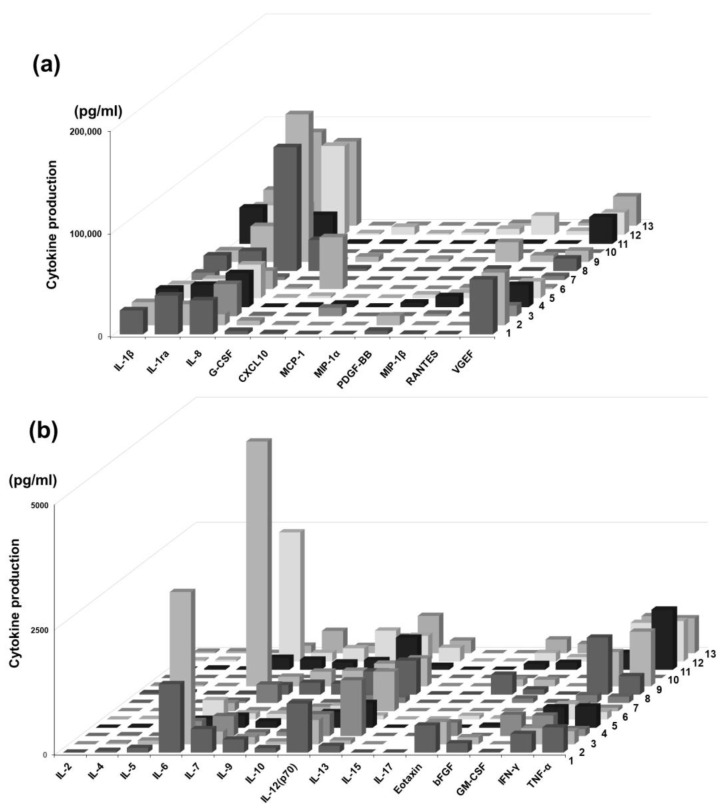
Cytokine profile of the apical exudate of patients. Cytokine concentrations in exudates derived from apical periodontitis were measured using the Bio-Plex^®^ system (Bio-Rad), following the manufacturer’s instructions. Different cytokines were detected from the periapical lesion samples at the initial visit or disease presentation. However, cytokine production varied among the cases. Especially, the production of IL-1β, IL-2, IL-4, IL-5, IL-6, IL-8, IL-10, IL-12, IL-13, IL-17, G-CSF, GM-CSF, IFN-γ, MCP-1, MIP-1α, MIP-1β, and TNF-α showed the difference. Comparison of cytokine profile indicated that cytokine production was variable before and after RCT (**a**,**b**).

Different cytokines were detected from the samples obtained from periapical lesions at the initial visit. However, cytokine production varied across patients. In particular, the production of IL-1β, IL-2, IL-4, IL-5, IL-6, IL-8, IL-10, IL-12, IL-13, IL-17, G-CSF, GM-CSF, IFN-γ, MCP-1, MIP-1α, MIP-1β, and TNF-α showed the difference among the patients. Based on these findings, patient profiles can be broadly divided into three patterns: inflammation-dominant (strong IL-1β and IL-8), repair/angiogenesis-dominant (strong VEGF and PDGF-BB), and chemokine-dominant (prominent MCP-1 and IP-10). The levels of several inflammatory cytokines, including IL-1β, IL-1ra, IL-6, IL-7, IL-8, IL-9, IL-10, IL-12(p70), IL-13, Eotaxin, bFGF, G-CSF, GM-CSF, IFN-γ, CXCL10, MCP-1, MIP-1α, PDGF-BB, RANTES, TNF-α, and VEGF, were markedly elevated. In contrast, the concentrations of MIP-1β, IL-2, IL-4, IL-5, IL-15, and IL-17 demonstrated little to no increase. Comparison of cytokine profiles indicated that cytokine production varied among the patients (Figure 1a,b). In particular, when comparing cytokine patterns between patients, IL-1β, IL-1ra, IL-8, MCP-1, and VEGF were found to be particularly high in many patients. These molecules represent inflammation (IL-1 system, IL-8), immune cell recruitment (MCP-1), and repair/angiogenesis (VEGF), and may be central to periapical lesions. Furthermore, many of the cytokines showing constant levels were Th1/Th2/Th17 cytokines, suggesting that the innate immune system and chemokine system, rather than T cell cytokines, may play a key role in the pathogenesis of periapical lesions.

### 3.2. Changes in Cytokine Profiling During Treatment

**Figure 2:**
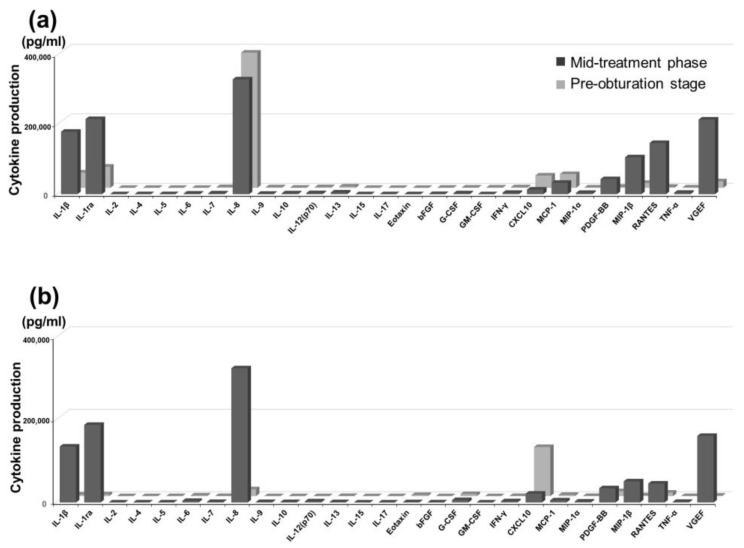
Changes in cytokine profile during treatment. Cytokine concentrations in exudates deriving from apical periodontitis were measured using the Bio-Plex^®^ system (Bio-Rad), following the manufacturer’s instructions. Cytokine levels in the apical exudates were compared between the mid-treatment phase and the stage immediately before root canal obturation for each patient. Marked inter-individual variability was observed in cytokine production, particularly in IL-1β, IL-1ra, IL-8, CXCL10, MCP-1, PDGF-BB, MIP-1α, RANTES, and VEGF. Notably, in patients who exhibited favorable healing, CXCL10 levels were elevated at the pre-obturation stage compared with the mid-treatment phase (**a**,**b**).

Cytokine levels in the apical exudates were compared between the mid-treatment phase and the time when sterilization was confirmed by bacterial testing for each patient. Marked inter-individual variability was observed in cytokine production, particularly in IL-1β, IL-1ra, IL-8, CXCL10, MCP-1, PDGF-BB, MIP-1β, RANTES, and VEGF. In particular, in patients who exhibited favorable healing, CXCL10 levels were elevated at the pre-obturation stage compared with those at the mid-treatment phase (Figure 2a,b).

### 3.3. Chemotactic Activity of Monocytes by CXCL10

**Figure 3:**
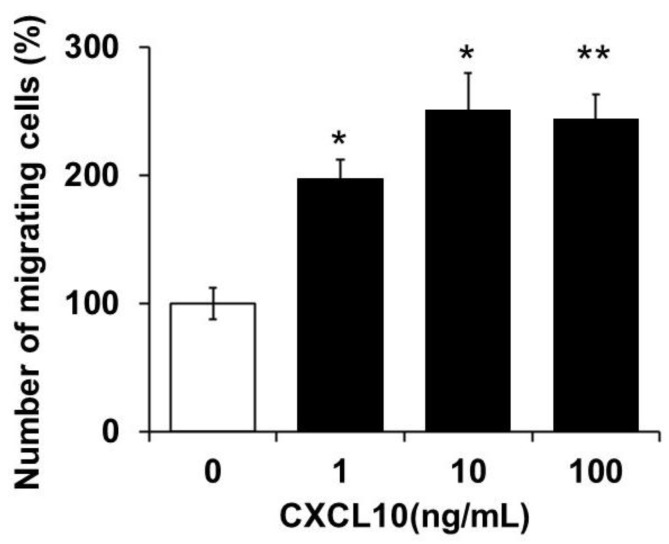
Chemotactic activity of monocytes by CXCL10. The addition of CXCL10 significantly enhanced the migration of BMMCs, with observed increases of 97.2 ± 15.4% at 1 ng/mL, 151.6 ± 27.9% at 10 ng/mL, and 144.1 ± 19.1% at 100 ng/mL. (* *p* < 0.01, ** *p* < 0.001; Student’s *t*-test).

Based on clinical findings, we focused on CXCL10, which was elevated in apical exudates of patients exhibiting favorable healing. Previous studies have reported that CXCL10 promotes metastasis and progression in cancer cells [11]. These findings led us to hypothesize that CXCL10 may similarly enhance the migratory behavior of BMMCs. To test this hypothesis, we investigated the effect of CXCL10 on the migration of BMMC. The addition of CXCL10 significantly improved the migration of BMMCs, with observed increases of 97.2 ± 15.4% at 1 ng/mL, 151.6 ± 27.9% at 10 ng/mL, and 144.1 ± 19.1% at 100 ng/mL (Figure 3).

## 4. Discussion

Cytokine profiling of apical exudates revealed a broad inflammatory response with marked inter-individual variability. At the initial visit or presentation, nearly all cytokines were detectable in the periapical exudates, although their levels varied considerably between patients. In particular, proinflammatory and chemotactic cytokines such as IL-1β, IL-6, IL-8, IL-10, IFN-γ, TNF-α, MCP-1, MIP-1α, and G-/GM-CSF were consistently elevated in many cases. In contrast, cytokines commonly associated with Th2 and Th17 responses—including IL-2, IL-4, IL-5, IL-15, and IL-17—showed minimal or no elevation (Figure 1a,b).

Longitudinal analysis revealed dynamic changes in cytokine expression during treatment. A comparison of the mid-treatment and pre-obturation stages demonstrated variable expression patterns, with particularly large shifts observed in IL-1β, IL-1ra, IL-8, CXCL10, MCP-1, MIP-1β, PDGF-BB, RANTES, and VEGF (Figure 2a,b).

Among them, CXCL10 was markedly elevated in patients with favorable healing outcomes, suggesting a potential role in the resolution phase of periapical inflammation. Functional assays further supported this finding, as CXCL10 significantly improved BMMCs migration in a dose-dependent manner (Figure 3), indicating that its chemotactic activity can contribute to tissue remodeling or immune regulation during healing.

Traditionally, the evaluation of periapical lesions has been heavily based on microbiological approaches, such as bacterial culture and antibiotic susceptibility tests, to identify pathogenic species and guide antimicrobial therapy [12,13,14]. However, culture-dependent methods are inherently limited: they often fail to capture the full microbial diversity—including fastidious or viable-but-non-culturable species—and may underestimate the prevalence of pathogens [15]. Nested polymerase chain reaction and molecular sequencing techniques have revealed persistent organisms, such as *Enterococcus faecalis* and *Porphyromonas gingivalis*, even after chemo-mechanical preparation and intracanal medication [14]. Consequently, newer strategies combining microbial culture with molecular assays (e.g., whole-genome amplification + polymerase chain reaction-based testing) are increasingly being advocated to achieve comprehensive detection and better inform treatment planning [16].

In the present study, we focused on host-derived factors, with particular attention to cytokine responses within periapical lesions. Although the microbiological aspects of AP—such as bacterial identification and antibiotic susceptibility—have been extensively studied, increasing evidence highlights the crucial role of host immune mediators, including cytokines and chemokines, in both the pathogenesis and resolution of periapical inflammation [17]. This notion is supported by previous reports that indicate that key inflammatory cytokines—such as IL-1β, IL-6, TNF-α, and CXCL10—are deeply involved in the progression and healing of periapical lesions [2].

CXCL10 was found to be elevated in patients exhibiting favorable healing following RCT, suggesting a potential role in immune-mediated tissue repair. However, these data do not definitively establish whether CXCL10 actively contributes to healing or merely reflects resolution of inflammation.

A key limitation of the present study is the small sample size of exudate specimens, which may not adequately capture the full heterogeneity of immune responses in periapical lesions. Furthermore, although our in vitro results demonstrated that CXCL10 promotes the migration of bone marrow–derived mononuclear cells BMMCs, the specific immune cell subsets involved in this chemotactic effect remain unclear.

There are also other limitations. First, the sample size was relatively small (*n* = 13), which limits the statistical power and generalizability of the findings. No sample size calculation or power analysis was conducted in advance, and the study should be considered exploratory in nature. Second, the inclusion of patients with heterogeneous diagnoses—acute apical periodontitis, chronic apical periodontitis, and radicular cysts—may have influenced the cytokine profiles. Due to the limited number of cases, subgroup analysis was not feasible, and all cases were analyzed collectively. Future studies with larger cohorts and longitudinal validation are warranted to allow stratified comparisons between disease types.

Notably, previous studies have reported both protective and pathogenic roles for CXCL10 in various chronic inflammatory conditions [2], suggesting that its impact may be context-dependent. These findings underscore the need for further studies to clarify the role of CXCL10 in the immunopathology and healing of periapical lesions.

Taken together, these findings highlight the potential importance of CXCL10 in the regulation of inflammatory cell recruitment and resolution processes within periapical lesions. Based on our results, it is plausible that CXCL10 contributes to favorable healing results by promoting monocyte migration and facilitating immune modulation at the site of inflammation. However, the results of this study only capture a phenomenon, and it is unclear whether the direct function of CXCL10 affects the healing of periapical lesions. For example, cytokine profiling in the exudates from periapical lesions at initial consultation could be used as a biomarker to evaluate the prognosis of root canal treatment, and administration of CXCL10 via the root canal or local administration of a CXCL10 inhibitor to the periapical lesion could potentially be used as a therapeutic agent. However, although in vitro studies have demonstrated that CXCL10 induces mononuclear cell migration, this is not sufficient. Further research, including in vivo models, is warranted to clarify its mechanistic role in periapical healing.

## 5. Conclusions

The findings of this study suggest that the cytokine profile of the periapical lesion might be useful for assessing the current condition of the periapical lesion.

## Figures and Tables

**Table 1:** Clinical profiles of patients.

Pt	Age	Sex	Site	Date	Diag.	Healing Outcomes	Systemic Comorbidities
1	49	♀	46	first visit	acute apical periodontitis	Healing of the periapical lesion	Cleft palate
2	43	♂	12	first visit	acute apical periodontitis or redicular cyst	Healing of the periapical lesion	
3	68	♂	22	first visit	acute apical periodontitis	Extraction	Pharyngeal cancer
4	53	♀	12	first visit	acute apical periodontitis	Healing of the periapical lesion	bronchial asthma
5	67	♀	24	first visit	acute apical periodontitis	Healing of the periapical lesion	cerebral infarction
6	58	♀	11	first visit	chronic apical periodontitis	Healing of the periapical lesion	diabetes mellitus
7	42	♂	26	first visit	chronic apical periodontitis or redicular cyst	Root resection	
8	82	♀	22	first visit	chronic apical periodontitis	Apicoectomy	rheumatoid arthritis
9	59	♂	16	first visit	chronic apical periodontitis	Healing of the periapical lesion	hyper tension
10	63	♂	36	first visit	chronic apical periodontitis	Healing of the periapical lesion	
11	79	♀	13	first visit	chronic apical periodontitis	Healing of the periapical lesion	diabetes mellitus, hyper tension
12	66	♀	13	first visit	chronic apical periodontitis	Healing of the periapical lesion	
13	69	♂	16	first visit	chronic apical periodontitis	Healing of the periapical lesion	stomach cancer

## Data Availability

All data generated or analyzed during this study are included in the published article.

## Abbreviations

The following abbreviations are used in this manuscript:

BMMC	bone marrow-derived mononuclear cells
IL	interleukin
G-CSF	granulocyte colony-stimulating factor
GM-CSF	granulocyte-macrophage colony-stimulating factor
IFN-γ	interferon gamma
MCP	monocyte chemoattractant protein
MIP	macrophage inflammatory protein
TNF	tumor necrosis factor
AP	apical periodontitis
RAP	refractory apical periodontitis
RCT	root canal treatment
